# Development of a Novel Diagnostic Support Tool for Degenerative Cervical Myelopathy Combining 10-s Grip and Release Test and Grip Strength: A Pilot Study

**DOI:** 10.3390/diagnostics12092108

**Published:** 2022-08-31

**Authors:** Hiroshi Kobayashi, Koji Otani, Takuya Nikaido, Kazuyuki Watanabe, Kinshi Kato, Yoshihiro Kobayashi, Shoji Yabuki, Shin-ichi Konno

**Affiliations:** Department of Orthopedic Surgery, School of Medicine, Fukushima Medical University, Fukushima 960-1295, Japan

**Keywords:** myelopathy, degenerative cervical myelopathy, cervical spondylotic myelopathy, ossification of posterior longitudinal ligament, cervical disc herniation, primary care, screening, 10-s grip and release test, grip strength

## Abstract

Early diagnosis of degenerative cervical myelopathy (DCM) is desirable, as delayed treatment can cause irreversible spinal cord injury and subsequent activity of daily living (ADL) impairment. We attempted to develop a straightforward and accurate diagnostic tool for DCM by combining the grip and release test (GRT) and grip strength. As a pilot study, we measured the GRT and grip strength of patients with DCM (n = 247) and a control group (n = 721). Receiver operating characteristic analysis was performed using the lower left and right. The Youden index was used to set cutoff values by sex and age group. The diagnostic performance of each test varied by sex and age, and a diagnostic support tool was created to determine any abnormal results in a test. The calculated M/F cutoff values for GRT were as follows: 40–59 years, 21/18; 60–69 years, 17/17; 70–79 years, 15/15; and 80–89 years, 11/12. The calculated M/F cutoff values for grip strength 32/20, 29/13, 21/15, and 19/10. When either GRT or grip strength was judged as positive, the overall sensitivity was 88.2%, specificity was 78.1%, positive likelihood ratio was 4.03, and the negative likelihood ratio was 0.15. This novel diagnostic support tool was superior to using GRT and grip strength alone in the early DCM diagnosis. Future research to obtain age- and sex-specific data is necessary to validate and further improve the tool.

## 1. Introduction

Degenerative cervical myelopathy (DCM) is the most common cause of spinal cord disorders [1], with a reported incidence of hospitalizations due to DCM ranging from 4.04 to 7.88 per 100,000 individuals [2,3] and surgery at 1.6 per 100,000 [4]. However, owing to a lack of a uniform definition and extensive population-based surveys, the exact prevalence is unknown, and the true prevalence is suspected to be much higher [5]. The prevalence of this syndrome is on the rise due to an aging society, and the number of surgeries associated with this disorder is increasing every year [6]. It is therefore expected that DCM will become a social disability burden [7]. 

DCM involves gradual deterioration of symptoms, but surgery at the appropriate time can help improve symptoms and activities of daily living (ADLs) [8]; early diagnosis is therefore desirable. Patients with early DCM often seek primary care, but it can take up to two years to establish the diagnosis. This delay may be attributable to a lack of knowledge of DCM signs in primary care and the need for awareness of DCM in the field of family medicine [9].

To date, various simple assessment methods for DCM diagnosis, which can potentially be performed in primary care settings, have been reported. We have previously reported a cutoff value by sex and age group for the diagnosis of DCM based on grip strength [10]. The 10-s grip and release test (GRT) is another common diagnostic indicator of cervical myelopathy [11]. These two tests evaluate the symptoms of the patient from different perspectives, such as muscle strength and lack of hand dexterity. Still, when used alone, their sensitivity and specificity vary by sex and age, limiting their usefulness. We hypothesized that the diagnostic performance can be improved by combining both tests, which may promote early diagnosis and surgery of patients with DCM, prevent a decline in ADL, and increase healthy life expectancy. 

In this study, with the aim of developing a highly accurate diagnostic support tool for DCM, we first carried out a pilot study to test this tool by simultaneously measuring GRT and grip strength in patients undergoing DCM surgery and in healthy controls.

## 2. Materials and Methods

### 2.1. Ethical Considerations

This study was approved by the Ethics Committee of our institution (No. 1880) and was conducted in accordance with the Declaration of Helsinki. Informed consent was obtained in the form of an opportunity to opt-out of participation on the institution’s website. 

### 2.2. Study Design

This single-center, cross-sectional study used prospectively collected data to validate diagnostic accuracy in patients with DCM. The DCM group comprised patients visiting our hospital, and the control group included individuals who had participated in a local government health checkup program.

### 2.3. Study Participants

#### 2.3.1. DCM Group

Patients with DCM aged 40–89 years who had undergone surgery at our hospital between May 2005 and April 2017 were consecutively included in the DCM group. Indications for surgery were determined by consensus between at least three board-certified spine surgeons. Patients with a history of cervical spine surgery, cervical trauma, pyogenic spondylitis, cerebrovascular disease, rheumatoid arthritis (RA), destructive spondyloarthropathy, atlanto-axial subluxation, pseudotumor of dens, cervical spondylotic amyotrophy, cerebral palsy, or malformation of the cervical spine were excluded.

#### 2.3.2. Control Group

The control group comprised individuals aged 40–89 years, who had provided written informed consent and wished to undergo a cervical spine health examination (interview and physical examination), from among those who participated in regular health examinations conducted in May, August, and November 2005 by local governments in the mountainous areas of Tadami Town, Ina Village, and Tateiwa Village of Fukushima Prefecture. The checkup was part of an epidemiological study conducted in 2005, involving local residents with musculoskeletal disorders [12]. Further, individuals with a history of cerebrovascular disease were excluded. Individuals with possible symptoms of myelopathy, such as hand numbness and maladroitness, were also excluded on the basis of interviews and physical examinations conducted by orthopedic surgeons to rule out individuals with a potential diagnosis of DCM. Specifically, individuals with signs and symptoms that included hand dysfunction, numbness in both hands, pain, or numbness radiating to the upper extremities, and spinal signs such as positivity for Jackson’s head compression test, the Spurling test, and Jackson’s shoulder depression test were excluded [13,14].

### 2.4. Examination of GRT and Grip Strength

In the DCM group, GRT and grip strength were measured the day before or on the day of surgery. In both groups, the measurements were performed by orthopedic surgeons.

For the GRT, patients and controls were seated in front of the examiner and instructed to grasp and open their fingers as quickly as possible, one side at a time, with the forearms kept in pronation and with mild wrist extension. The number of complete movement cycles performed in 10 s was counted for each side separately [15]. Patients unable to fully grip or open their fingers were asked to perform this action as much as possible. The grip strength was measured in the standing position for patients and controls who could stand and in the sitting position for those who could not. The upper limbs were held in a drooped position and measured using a Dynamometer EKJ080 (Evernew, Tokyo, Japan). This grip strength meter is of the analog type and can measure every 0.5 kg.

For both GRT and grip strength tests, the lower value from among the results of the two sides was used. This method has been used in the development of grip strength screening methods and has been reported to be valid [10].

### 2.5. Statistical Analyses

The mean values for GRT and grip strength in both hands were compared between the two groups in terms of sex and age using a Wilcoxon rank-sum test, and *p*-values < 0.05 were considered statistically significant. Receiver operating characteristic (ROC) curve analysis was performed. The area under the curve (AUC) was calculated. 

The Youden index was then used to set cutoff values for the two tests [16]. The sensitivity, specificity, positive likelihood ratio, and negative likelihood ratio were calculated when each test was used alone. Finally, a support tool was created to determine positive results for GRT or grip strength abnormalities. Its sensitivity, specificity, positive likelihood ratio, and negative likelihood ratio were calculated.

All statistical analyses were performed using JMP Pro software program version 15.0.0 (JMP Statistical Discovery, Cary, NC, USA).

## 3. Results

**Figure 1:**
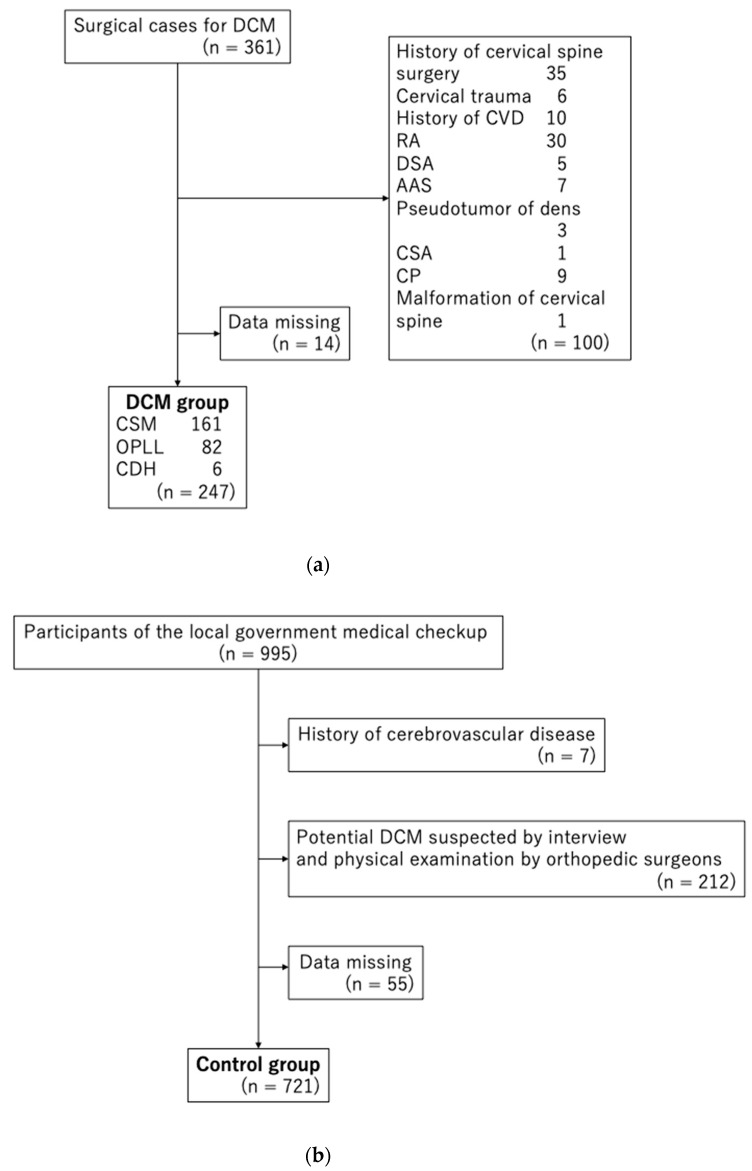
(**a**). Flow chart showing recruitment of the DCM group. DCM, degenerative cervical myelopathy; CVD, cardiovascular disease; RA, rheumatoid arthritis; DSA, destructive spondyloarthropathy; AAS, atlanto-axial subluxation; CSA, cervical spondylotic amyotrophy; CP, cerebral palsy; CSM, cervical spondylotic myelopathy; OPLL, ossification of posterior longitudinal ligament; CDH, cervical disc herniation. (**b**). Flow chart showing recruitment of the control group. DCM, degenerative cervical myelopathy.

We excluded 100 of 361 participants from the DCM group, including those with a history of cervical spine surgery (n = 35), cervical trauma (n = 6), cerebrovascular disease (n = 10), RA (n = 30), destructive spondyloarthropathy (n = 5), atlanto-axial subluxation (n = 7), pseudotumor of dens (n = 3), cervical spondylotic amyotrophy (n = 1), cerebral palsy (n = 9), and malformation of the cervical spine (n = 1), along with duplicates. Moreover, we excluded 14 participants with missing GRT and/or grip strength data. Eventually, we analyzed 247 participants (165 men, 82 women; largest age group, 70–79 years; and mean age, 65.2 years) (Figure 1a). The DCM group comprised 161, 82, and 6 participants with cervical spondylotic myelopathy, ossification of the posterior longitudinal ligament, and cervical disc herniation, respectively.

We excluded seven of 995 participants from the control group because of a history of cerebrovascular disease. We excluded 212 participants because the potential diagnosis of DCM could not be eliminated based on interviews and physical examinations. Moreover, 55 participants were excluded because of missing grip strength data. Eventually, we analyzed 721 participants (275 men, 446 women; largest age group, 70–79 years; and mean age, 65.9 years) (Figure 1b). 

The mean values of the GRT in the DCM group in terms of the sex (men/women (M/F)) and age (40–59 years, 60–69 years, 70–79 years, and 80–89 years; ±SD) were M/F 18.5 ± 8.2/16.6 ± 5.3, 13.9 ± 6.0/12.5 ± 4.8, 15.0 ± 5.4/12.2 ± 3.9, and 11.4 ± 4.7/9.5 ± 3.8 times, respectively, whereas those in the control group were M/F 27.0 ± 5.6/23.5 ± 4.8, 23.5 ± 4.7/20.7 ± 4.7, 20.2 ± 5.2/19.2 ± 4.8, and 20.1 ± 5.0/17.8 ± 4.3, respectively (Table 1a). The mean values of grip strength in the DCM group in terms of the sex and age were M/F 23.3 ± 12.1/14.5 ± 7.4, 16.5 ± 9.1/10.0 ± 6.2, 16.5 ± 8.0/8.5 ± 4.9, and 10.8 ± 6.6/4.4 ± 4.0, respectively, whereas those in the control group were M/F 42.6 ± 7.7/25.6 ± 6.8, 35.5 ± 7.8/20.3 ± 4.6, 30.1 ± 7.5/17.6 ± 4.8, and 26.0 ± 5.7/15.2 ± 3.8, respectively (Table 1b).

### ROC Analyses

The AUC of the GRT in terms of the sex (men/women (M/F) and age (40–59 years, 60–69 years, 70–79 years, and 80–89 years) were M/F 0.82/0.84, 0.91/0.91, 0.75/0.87, and 0.90/0.92, respectively (Table 2a), whereas that of grip strength was 0.92/0.87, 0.94/0.91, 0.89/0.91, and 0.97/0.97, respectively (Table 2b). 

The cutoff values for the GRT using the Youden index in terms of the sex (men/women (M/F) and age (40–59 years, 60–69 years, 70–79 years, and 80–89 years) were M/F 21/18, 17/17, 15/15, and 11/12, respectively (Table 2a), whereas those for grip strength were 32/20, 29/13, 21/15, and 19/10, respectively (Table 2b). A measured value equal to or less than the cutoff value was considered abnormal. Table 2a,b summarize the sensitivity, specificity, positive likelihood ratio, and negative likelihood ratio by the sex and age group upon using the respective cutoff values for the GRT and grip strength. For all sex and age groups combined, the sensitivity, specificity, positive likelihood ratio, and negative likelihood ratio for the GRT and grip strength were 0.73, 0.83, 4.31, and 0.33, and 0.83, 0.86, 5.78, and 0.19, respectively.

We combined these results to create a diagnostic support tool that determined the abnormalities for either the GRT or grip strength at or below the cutoff value (Table 3). The sensitivities and specificities of this DCM diagnostic support tool for the sex (men/women (M/F) and age (40–59 years, 60–69 years, 70–79 years, and 80–89 years) were M/F 0.90/0.91, 0.96/0.94, 0.79/1.00, and 0.92/0.93, and 0.82/0.77, 0.74/0.70, 0.78/0.56, and 0.94/0.83, respectively. The positive- and negative likelihood ratios for the sex (men/women (M/F) and age (40–59 years, 60–69 years, 70–79 years, and 80–89 years) were 4.94/4.01, 3.71/3.20, 3.65/2.26, and 15.58/5.47, and 0.12/0.12, 0.06/0.08, 0.27/0.00, and 0.09/0.08, respectively. Upon calculating all sex and age groups together, the sensitivity, specificity, positive-, and negative likelihood ratio was 0.91, 0.73, 3.37, and 0.12, respectively (Table 4).

## 4. Discussion

### 4.1. Short Summary

In this study, we attempted to create a diagnostic support tool having a superior diagnostic ability based on data pertaining to GRT and grip strength measurements in healthy control and DCM surgery groups. We found that GRT and grip strength exhibited moderate or high diagnostic accuracy individually, with AUCs ≥ 0.70 for all sex and age groups [17]. The Youden index was used to determine the cutoff values for GRT and grip strength individually for DCM diagnosis according to sex and age groups [16]. However, the sensitivity and specificity of both tests differed according to sex and age groups, thus making it difficult to apply them in clinical practice without the clinical staff having a clear understanding of the tests’ associated characteristics. Therefore, we created a diagnostic support tool that defines a positive result when either of these two cutoff values is abnormal, thereby improving diagnostic performance. 

As a result, we improved the sensitivity of the test in all sex and age groups. The specificity and positive and negative likelihood ratios were good, confirming that the diagnostic support tool that we developed is both easy to use and diagnostically valuable.

Multiple mechanisms are involved in the development of cervical myelopathy, including static [18] and dynamic factors [19,20] and ischemia [21], and the initial symptoms may differ depending on the site of occurrence. These varied mechanisms may be a reason for the differing diagnostic performance of single-criteria assessments. We used both GRT and grip strength in this study because they assess slightly different mechanisms of hand clumsiness and muscle strength, respectively, and the measurements are simple. The results of this study suggest that although DCM has a variety of pathogenic mechanisms, most cases are caused by impaired fine motor skills or muscle strength in the early stages.

### 4.2. Diagnosis of DCM

There is no gold standard for diagnosing DCM, and it is usually diagnosed on the basis of physical findings consistent with imaging results. The initial diagnosis of DCM using physical findings alone is often difficult: Glaser et al. reported a positivity rate of 58% for the Hoffmann test in patients with spinal cord compression evident on magnetic resonance imaging (MRI) [22]; Chaiyamongkol et al. reported a sensitivity of 95% for the Trömner reflex in patients with spinal cord compression evident on MRI [23]. However, the actual pathological significance of these reflexes in outpatients is unknown because these reports do not include comparisons with data obtained from healthy participants. False positive results for these reflexes can also occur due to the presence of intracranial lesions or hyperreflexia.

Nagata et al. compared the cervical cord compression and no compression groups using MRI in health examination participants. They reported that the results of GRT and lower extremity function test were significantly associated with cervical cord compression [24]. Similarly, Hirai et al. compared the cervical cord compression and no compression groups using MRI in health examination participants [25]. They found that the following were not associated with spinal cord compression evident on MRI: Hoffmann, Trömner, and Wartenberg, Babinski, and Chaddock signs; hypersensitivity of the patellar and Achilles tendon reflexes; ankle clonus; Romberg and modified Romberg tests; GRT; finger escape sign; and grip strength. These two studies performed in community residents indicate that the compression that was evident on MRI cannot be diagnosed using a simple physical examination alone. 

In a previous systematic review, Singh et al. compared the results of various clinical tests in patients with DCM and healthy controls and further reviewed the validity, reliability, and responsiveness of clinical tests, such as GRT, 30-m walking test, triangle step test, foot-tapping test, gait velocity, steps, step length, step width, stance phase duration, single stance-phase duration, stance phase, double support, flexion of the knee, plantar flexion of the ankle, and nine-hole peg test [26]. They are commonly used to quantify the degree of impairment; however, determining the cutoff values for the measurement results may aid in the diagnosis. Machino et al. reported a cutoff value for GRT in the diagnosis of DCM [11]. We have also reported our attempts to screen for DCM by establishing cutoff values according to sex and age groups for grip strength [10]. However, these reports had variable sensitivity and specificity according to sex and age group, complicating the interpretation of the results.

MRI is the standard in DCM imaging; MRI can help in assessing the nerves, bones, and ligament structures with high resolution [27]. However, as mentioned above, spinal cord compression evident on imaging does not always correspond with the symptoms [24,25]. Recently, quantitative MRI and 3 T-MRI have been reported to detect spinal cord tissue damage and are expected to exhibit improved diagnostic performance [28]. However, since MRI facilities are limited, it is still difficult to determine the need for performing MRI.

In recent years, advances in digital grip dynamometers have made it possible to collect and observe isometric grip forces in real-time and to measure muscle functional characteristics other than maximal force, such as force development, submaximal force stability, and fatigability [29]. These parameters are potential candidates for DCM screening tools.

In addition, smartphones, wearable technology, and artificial intelligence have been developed to serve as new diagnostic tools for DCM [30,31]. However, they have not yet been widely used in clinical practice. At this point, further accumulation of knowledge is needed regarding the items that should be used as indicators to create an effective diagnostic tool.

### 4.3. Advantages and Cautions in the Use of the DCM Diagnostic Support Tool

The first advantage of the novel diagnostic support tool for DCM is that it can be used quickly with a timer, grip dynamometer, and cutoff value table (Table 3). In fact, since grip strength meters are commonly used in internal medicine, such as in the diagnosis of sarcopenia [32], they can be easily introduced in primary care. In recent years, various educational videos for the performance of physical examinations have become available, and such videos can make learning the method of performing GRT more accessible for nonspecialists [33]. Second, because the tool is simple and noninvasive, it can be used by paramedical staff to obtain measurements before a medical visit, thereby shortening the duration of actual medical examination. In the future, the tool could be used in screening for DCM in medical checkup camps and self-assessment. However, its usefulness in healthy subjects should be investigated. Third, this tool can be helpful in follow-up if the trends of values obtained in patients suspected of having DCM are recorded. The available time-course can also help surgeons in deciding the time for surgery while referring patients to advanced medical institutions. In addition, postoperative checkups can provide an accurate picture of improvement, which can be shared with the patient.

Despite these advantages, several points should be kept in mind while using this tool. First, the tool shows good sensitivity for most sex and age groups, but its sensitivity for men in their 70 s is 0.79; this limitation must not be overlooked. Second, grip strength can be compromised in distal radius fracture, peripheral neuropathy, lateral humeral epicondylitis, and arthritis of the carpometacarpal joint [34]. In addition, cases that develop lower extremity symptoms may be missed. Nagata et al. found that asymptomatic patients with spinal cord compression on MRI who developed the disease four years later had increased exacerbations of lower extremity symptoms [35]. Therefore, this tool should be viewed as a support tool only, and examining doctors should be encouraged to identify larger numbers of suspected patients and refer them to a specialist.

### 4.4. Limitations

There are several limitations to this study. First, most included patients were male, while the healthy control group comprised predominantly females. This could be because, epidemiologically, DCM occurs predominantly in males [36], and since participants in the healthy control group were recruited from national health insurance screening, males often participated in workplace checkups instead of the national health insurance checkups, resulting in a lower proportion of males in the healthy control group. Although a matched-pairs study could not be performed owing to these limitations, this issue was addressed by analyzing the data according to sex and age groups.

Second, all patients in this study were treated surgically. This point implies that we discriminated patients with DCM that required surgical treatment to prevent and improve ADL impairment through surgery.

Third, racial differences and body size should be considered before generalizing the results of this study: concerning DCM, Asians have a higher prevalence of ossification of posterior longitudinal ligament (OPLL) [37], and the DCM group in this study may have a greater number of OPLL cases. The Japanese population is reported to have a smaller anterior–posterior spinal canal diameter than the average diameter in the Western population [38]. Grip strength has also been reported to be affected by body mass index [39]. 

Finally, since the healthy control group included participants from a rural area, future validation in urban residents is needed to generalize the results.

Despite these limitations, the present pilot study compared healthy and postoperative patients with DCM using this test during physical examinations performed by surgeons and reported a high diagnostic performance. The cutoff values by sex and age identified in this study need further data for validation, especially for the 80 s age group, owing to the small number of cases in the surgery group. We plan to present the final version of the tool in the near future by accumulating more data and further validating and improving the criteria.

## 5. Conclusions

This pilot study presents a DCM diagnostic support tool and demonstrates its high diagnostic performance. Further validation and improvement of this tool are planned with additional data. This DCM diagnostic support tool may be used in the initial diagnostic evaluation of patients with suspected DCM to prevent oversights and objectively determine a course of action.

## Figures and Tables

**Table 1:** (**a**)**.** Grip and release test by sex and age in both groups. (**b**). Grip strength by sex and age in both groups.

**(a)**						
	**Age (y)**	**n**		**GRT (Times)**		***p*-Value**
		**CTRL**	**DCM**	**CTRL**	**DCM**	
Male	40–59	82	62	27.0 ± 5.6	18.5 ± 8.2	<0.0001
	60–69	62	48	23.5 ± 4.7	13.9 ± 6.0	<0.0001
	70–79	97	43	20.2 ± 5.2	15.0 ± 5.4	<0.0001
	80–89	34	12	20.1 ± 5.0	11.4 ± 4.7	<0.0001
Female	40–49	119	22	23.5 ± 4.8	16.6 ± 5.3	<0.0001
	60–69	132	18	20.7 ± 4.7	12.5 ± 4.8	<0.0001
	70–79	154	27	19.2 ± 4.8	12.2 ± 3.9	<0.0001
	80–89	41	15	17.8 ± 4.3	9.5 ± 3.8	<0.0001
**(b)**						
	**Age (y)**	**n**		**Grip Strength (kg)**		***p*-Value**
		**CTRL**	**DCM**	**CTRL**	**DCM**	
Male	40–59	82	62	42.6 ± 7.7	23.3 ± 12.1	<0.0001
	60–69	62	48	35.5 ± 7.8	16.5 ± 9.1	<0.0001
	70–79	97	43	30.1 ± 7.5	16.5 ± 8.0	<0.0001
	80–89	34	12	26.0 ± 5.7	10.8 ± 6.6	<0.0001
Female	40–49	119	22	25.6 ± 6.8	14.5 ± 7.4	<0.0001
	60–69	132	18	20.3 ± 4.6	10.0 ± 6.2	<0.0001
	70–79	154	27	17.6 ± 4.8	8.5 ± 4.9	<0.0001
	80–89	41	15	15.2 ± 3.8	4.4 ± 4.0	<0.0001

GRT: grip and release test; CTRL: control; DCM: degenerative cervical myelopathy. CTRL: control; DCM: degenerative cervical myelopathy.

**Table 2:** (**a**). Area under the curve for the grip and release test in the screening of DCM. (**b**). Area under the curve for the grip test in the screening of DCM.

**Sex**		**AUC**	**Cutoff by Youden Index (Times)**	**Sensitivity**	**Specificity**	**LR (+)**	**LR (−)**
Male	40–59	0.82	21	0.68	0.85	4.53	0.38
	60–69	0.91	17	0.79	0.90	7.90	0.23
	70–79	0.75	15	0.58	0.85	3.87	0.49
	80–89	0.90	11	0.67	1.00	#DIV/0! ^†^	0.33
Female	40–49	0.84	18	0.73	0.89	6.64	0.30
	60–69	0.91	17	0.94	0.64	2.61	0.09
	70–79	0.87	15	0.78	0.75	3.12	0.29
	80–89	0.92	12	0.87	0.93	12.43	0.14
Total				0.73	0.83	4.31	0.33
(a)							
**Sex**		**AUC**	**Cutoff by Youden Index (kg)**	**Sensitivity**	**Specificity**	**LR (+)**	**LR (−)**
Male	40–59	0.92	32	0.68	0.85	11.57	0.20
	60–69	0.94	29	0.96	0.81	5.05	0.05
	70–79	0.89	21	0.70	0.91	7.78	0.33
	80–89	0.97	19	0.83	0.94	13.83	0.18
Female	40–49	0.87	20	0.73	0.82	4.06	0.33
	60–69	0.91	13	0.78	0.96	19.50	0.23
	70–79	0.91	15	0.96	0.71	3.31	0.06
	80–89	0.97	10	0.93	0.90	9.30	0.08
Total				0.83	0.86	5.78	0.19
(b)							

If the measured value is equal to or less than the cutoff value, it is determined to be abnormal. AUC: area under the curve; GRT: grip and release test; CTRL: control; DCM: degenerative cervical myelopathy; LR (+): positive likelihood ratio; LR (−): negative likelihood ratio. ^†^ #DIV/0! indicates that the denominator of LR+ is 0 because the specificity is 1.00.

**Table 3:** A novel diagnostic support tool for degenerative cervical myelopathy.

		Cutoff	
Sex		GRT (Times)	Grip Strength (kg)
Male	40–59	21	32
	60–69	17	29
	70–79	15	21
	80–89	11	19
Female	40–49	18	20
	60–69	17	13
	70–79	15	15
	80–89	12	10

If the measured value is equal to or less than the cutoff value, it is determined to be abnormal. If either GRT or grip strength readings are equal to or lower than the cutoff value, the system determines that it is abnormal. GRT: grip and release test.

**Table 4:** Diagnostic characteristics of the screening tool combining grip and release test and grip strength.

Sex	Age Group (y)	Sensitivity	Specificity	LR (+)	LR (−)
Male	40–59	0.90	0.82	4.94	0.12
	60–69	0.96	0.74	3.71	0.06
	70–79	0.79	0.78	3.65	0.27
	80–89	0.92	0.94	15.58	0.09
Female	40–59	0.91	0.77	4.01	0.12
	60–69	0.94	0.70	3.20	0.08
	70–79	1.00	0.56	2.26	0.00
	80–89	0.93	0.83	5.47	0.08
Total		0.91	0.73	3.37	0.12

LR (+): positive likelihood ratio; LR (−): negative likelihood ratio. If the measured value is equal to or less than the cutoff value, it is determined to be abnormal.

## Data Availability

Was obtained in the form of opt-out on the website.
